# Confidence is higher in touch than in vision in cases of perceptual ambiguity

**DOI:** 10.1038/s41598-018-34052-z

**Published:** 2018-10-23

**Authors:** Merle T. Fairhurst, Eoin Travers, Vincent Hayward, Ophelia Deroy

**Affiliations:** 1 Centre for the Study of the Senses, School of Advanced Study, University of London, London, UK; 2 Munich Center for Neuroscience, Ludwig Maximilian University, Munich, Germany; 3 Faculty of Philosophy, Ludwig Maximilian University, Munich, Germany; 4 Institute of Cognitive Neuroscience, University College London, London, UK; 5 Sorbonne Université, Institut des Systèmes Intelligents et de Robotique (ISIR), F-75005, Paris, France

## Abstract

The inclination to touch objects that we can see is a surprising behaviour, given that vision often supplies relevant and sufficiently accurate sensory evidence. Here we suggest that this ‘fact-checking’ phenomenon could be explained if touch provides a higher level of perceptual certainty than vision. Testing this hypothesis, observers explored inverted T-shaped stimuli eliciting the Vertical-horizontal illusion in vision and touch, which included clear-cut and ambiguous cases. In separate blocks, observers judged whether the vertical bar was shorter or longer than the horizontal bar and rated the confidence in their judgments. Decisions reached by vision were objectively more accurate than those reached by touch with higher overall confidence ratings. However, while confidence was higher for vision rather than for touch in clear-cut cases, observers were more confident in touch when the stimuli were ambiguous. This relative bias as a function of ambiguity qualifies the view that confidence tracks objective accuracy and uses a comparable mapping across sensory modalities. Employing a perceptual illusion, our method disentangles objective and subjective accuracy showing how the latter is tracked by confidence and point towards possible origins for ‘fact checking’ by touch.

## Introduction

**Figure 1:**
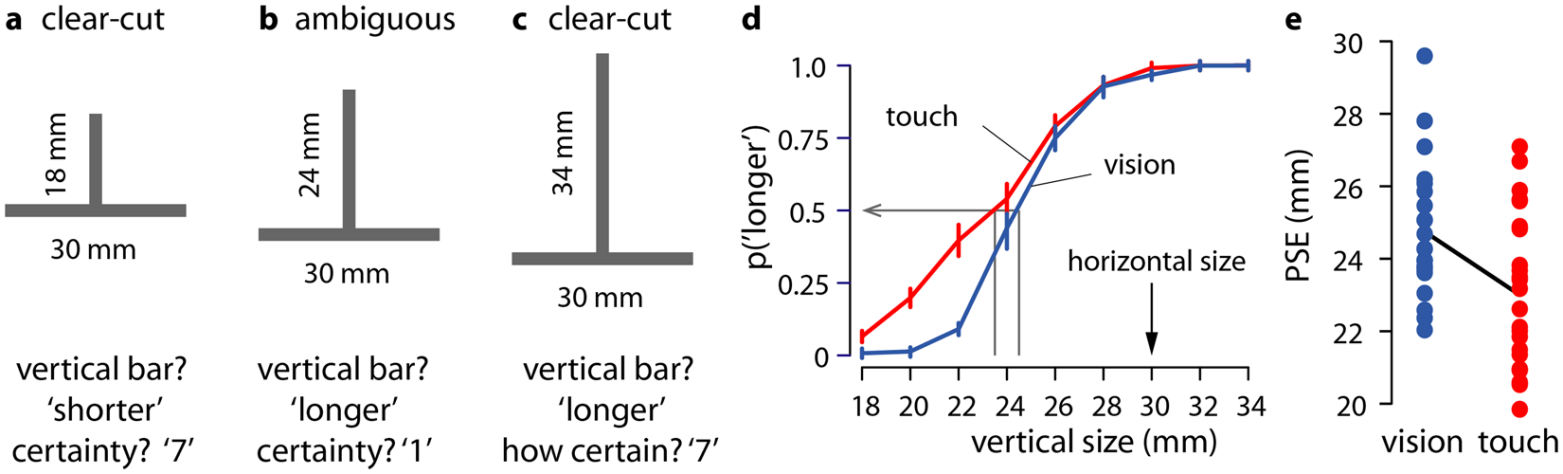
The Vertical-Horizontal Illusion (**a–c**) showing Inverted T stimuli were explored by touch and by vision. The vertical bar ranged from 18 to 34 mm, which was compared to a horizontal bar of fixed size of 30 mm. Stimuli could be clear-cut (**a**,**c**) or ambiguous (**b**) in the two modalities. Perceptual responses were verbal reports as to whether the vertical bar was ‘shorter’ or ‘longer’ than the horizontal bar. Confidence judgements were given on a scale from very uncertain (1) to very certain (7). (**a**) Probability of judging the vertical bar to be longer than the horizontal bar as a function of the size of the vertical bar. Error bars show within-participant standard errors. (**b**) Point of subjective equality (PSE) for each participant in each modality.

**Figure 2:**
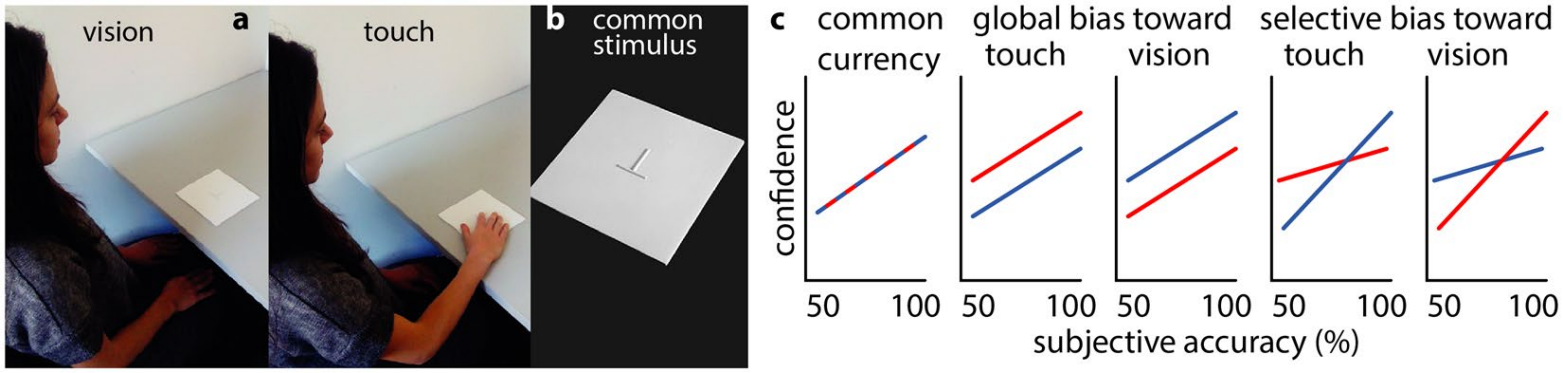
Stimuli and hypotheses (**a**) Testing conditions. (**b**) Stimulus designed to be clearly seen and felt. Hypothetical confidence profiles as a function of the difference from the PSE. (**c**) Possible confidence profiles: Common currency, global overconfidence in touch, global overconfidence in vision, greater confidence in touch under ambiguity, greater confidence in vision under ambiguity.

From museum visitors feeling compelled to touch statues that they can see, to the biblical account of the incredulous Thomas who would not accept that Jesus was alive unless he could touch him, tactile ‘fact-checking’ is frequent. Similarly, in the clinical domain, the empirical literature shows that individuals with obsessive compulsive disorder are prone to check things by touch rather than sight^1,2^. Among other factors underlying these complex behaviours, we suggest that the privilege of touch might come from it carrying more evidential weight than seeing particularly when there is ambiguity^3^. To test this hypothesis, we compared the confidence that observers put in their perceptual decisions after either seeing or touching stimuli that gave rise to a geometric illusion known as the Vertical-Horizontal (VH) illusion (Figs 1, 2a,b). This illusion is known to produce similar perceptual effects in the visual and in the tactile domains^4–6^.

The belief that touch provides more certainty than other senses, especially vision, has a solid historical background, but to our knowledge has not been directly tested, except with affordances^7^. Descartes, a sceptic toward all sensory evidence, highlighted that “Of all our senses, touch is the one considered least deceptive and the most secure”^8^ while Johnson, in response to Berkeley’s immaterialism^9^, considered that touch demonstrated the existence of an external world in a way that no other sense would (see also de Condillac for a similar claim^10^). The idea is that touch, more than vision, provides evidence for the reality of external objects^11,12^ and conveys a higher sense of directness and certainty^13,14^.

When it comes to providing evidence about certain features rather than the existence of objects, however, it is implausible that touch provides more objective or more accurate information than vision, since the relative accuracy of the two modalities depends critically on the task and on the context. There is a more sensible way of understanding the superiority of touch in this context: For equal accuracy, people might place more confidence in a decision reached by touch rather than vision. This hypothesis is congruent with evidence that people are more likely to purchase an item if they can touch it rather than if they simply look at it^15,16^, that some people are anxious when interacting with graphical user interfaces that display objects that cannot be touched^17,18^.

The study of confidence falls within the field of metacognition, i.e. how the cognitive system assesses and monitors its own states^19–21^. Studies comparing perceptual confidence across sensory modalities have been conducted previously but for tasks where each modality could not be directly compared, i.e. in a brightness versus pitch discrimination^22^, or orientation versus pitch discrimination. According to a widespread account of perceptual metacognition, a central purpose of explicit judgements of confidence is to allow the reliability of perception across different decisions to be compared, and appropriate trust to be placed in each percept accordingly.

The idea that confidence operates as a common currency at a given time across sensory modalities such as vision and audition has been directly tested by showing that people can determine which of two decisions should be trusted more both in the same modality as well as in different modalities^23,24^. By extension, the common currency model may extend through different decisional times by assuming that performance is mapped onto confidence in an identical manner across modalities. Decisions reached through different channels or in different contexts could then be compared. An ideal observer should then be able to decide which of two independent decisions to trust more, based on these comparable confidence ratings. This is this common mapping assumption that we tested across touch and vision.

If an observer makes correct decisions about three times out of four, both in vision and touch, and if confidence follows a common mapping, we would expect the observer to report the same confidence in her decisions, no matter what modality is used to arrive at a judgment, so long as the probable correctness of her decisions remains the same. If she was only correct two times out of four when relying on touch, and three out of four when relying on vision, she should report lower confidence for touch than vision. These two ratings would mean that she should choose to rely on vision, rather than touch. In other words, for confidence to be comparable between decisions, observers’ confidence ratings are expected to track the probability of their response being correct for a given stimulus, regardless of the modality used: they should express similar confidence when making decisions similarly likely to be correct, and different confidence when differently likely to be correct. Though individuals differ in their mappings from correctness to confidence, both in bias and sensitivity, a common mapping is indeed observed in the same individual across independent tasks and comparable sensitivities^22^. It is then likely to subserve the use of confidence as a common currency in direct comparisons.

There are several of ways in which the behavior of observers could depart from these assumptions (Fig. 2c). They could simply be over- or under-confident in one modality, causing them to rely on the corresponding sense more than they ought to. Alternatively, metacognitive sensitivity could differ between modalities as a result of the mappings between the perceptual accuracy (probability of being correct) and reported confidence.

Until now, few studies have compared perceptual metacognition in a task which can be performed by different sensory modalities^25^. While Fitzpatrick and colleagues looked at whether vision and touch would provide similar confidence in perceiving affordances for action, the task did not offer a fair comparison between the two modalities as a tool needed to be used for haptic exploration^7^. Furthermore, the perception of possibilities for action did not allow for an assessment of how confidence tracked accuracy. Here we chose to focus on size estimation, as providing a more straightforward domain in which to assess possible differences between tactile and visual confidence. We frequently use both vision and touch to estimate and compare the size of objects, and both senses are well suited to the task. Here, we focus on cases where the observer must decide between two options when sensory evidence is ambiguous (equivocal for both options). We investigated how size estimation in both vision and touch was affected by the robust Vertical-Horizontal illusion^4,26–28^ where a vertical bar appears to be longer than an adjoining horizontal bar of same length (see Fig. 1a–c). Studying metacognition across modalities using this task provides an intriguing opportunity to explore how confidence relates to observers’ subjective representations of the stimuli they perceive and follows a common mapping across two distinct tasks.

Observers explored the stimuli by vision and by touch in two separate testing blocks (Fig. 2a). In each case, they reported whether the vertical bar seemed to be longer or shorter than the horizontal bar. They were then asked to report how confident they were of their choice. Some stimuli were close to the point of subjective equality (PSE), such that the two bars appeared to be of the same size. This point corresponds to the case, discussed above, where there is equivocal evidence for both responses. Across both modalities, this was the case when the horizontal bar was approximately 25% longer than the vertical^29,30^. Accounting for this bias, our stimulus set varied the objective size of the vertical bar to include clear-cut cases where it was easy to determine whether the vertical size was shorter or longer than the horizontal one, even under the influence of the illusion. The stimulus set also included ambiguous cases where the illusion caused bars of different sizes to appear to have similar sizes, see Fig. 1. We expected that perceptual decisions in ambiguous cases, i.e. closer to the PSE, would be associated with lower confidence ratings for both modalities.

By pitting the two senses against one another in the fairest conditions possible over a range of ambiguous and clear-cut cases, this experiment could make a distinction between possible types of modality-related biases, sketched in Fig. 2c. If the common mapping assumption is correct, confidence should track perceived stimulus ambiguity in the same way across modalities. Alternatively, greater confidence might be placed in one or other modality overall, or, selectively, greater confidence in one or other modality under situations of certainty or uncertainty, suggesting a need to qualify the idea of a similar mapping. This in turns has broader implications for the generalization of common currency accounts, and whether confidence is assessed according to a similar and consistent metric across modalities, either when the decisions are compared immediately at the time of performance, or more generally for later purposes.

## Results

### Perceptual judgements

The proportion of observer responses stating that the vertical bar was longer than the horizontal reference bar, as a function of modality and of the vertical bar size, is plotted in Fig. 1d. The results demonstrated a robust perceptual bias in both modalities: observers almost unanimously stated that the vertical bar was longer than the horizontal when they were of the same size, and the sizes at which they were equally likely to give either response were considerably shorter than 30 mm. Psychometric functions fit for each observer, in each modality (see Methods) showed that perceptual sensitivity, S, was significantly greater for vision, S = 0.67, SD = 0.5, than for touch, S = 0.21, SD = 0.27, t(22) = 3.836, p < 0.001, d = 0.80. As shown in Fig. 1e, the PSE was significantly below 30 mm for vision, PSE = 24.6 mm, SD = 1.8, t(22) = 14.434, p < 0.001, d = 3.0, and for touch, PSE = 23.1 mm, SD = 2.1, t(22) = 15.766, p < 0.001, d = 3.3. The PSE was also slightly lower (further from 30 mm) for touch (23.1 mm) than for vision (24.6 mm), t(22) = 3.299, p = 0.003, d = 0.69. Thus, compared to touch, vision was better able to discriminate between different stimulus lengths, and was less subject to the Vertical-Horizontal illusion. Within modalities, greater sensitivity was associated with PSEs closer to the true value of 30 mm in touch, r(21) = 0.61, p = 0.002, but not in vision, r(21) = 0.20, p = 0.368.

### Confidence judgements

Across all trials, the observers’ confidence ratings, on a scale from 1 to 7, were significantly higher for vision, mean = 5.49, SD = 1.62, than for touch, mean = 4.99, SD = 1.55, t(22) = 3.263, p = 0.004, d = 0.7. The standard deviation of the confidence ratings for each observer did not differ significantly between vision, mean = 0.88, SD = 0.47, and touch, mean = 0.66, SD = 0.49, t(22) <1, p >0.3, d = 0.2. The distributions of the confidence ratings in each modality are reported in Supplementary Material.

### Confidence and Ambiguity

**Figure 3:**
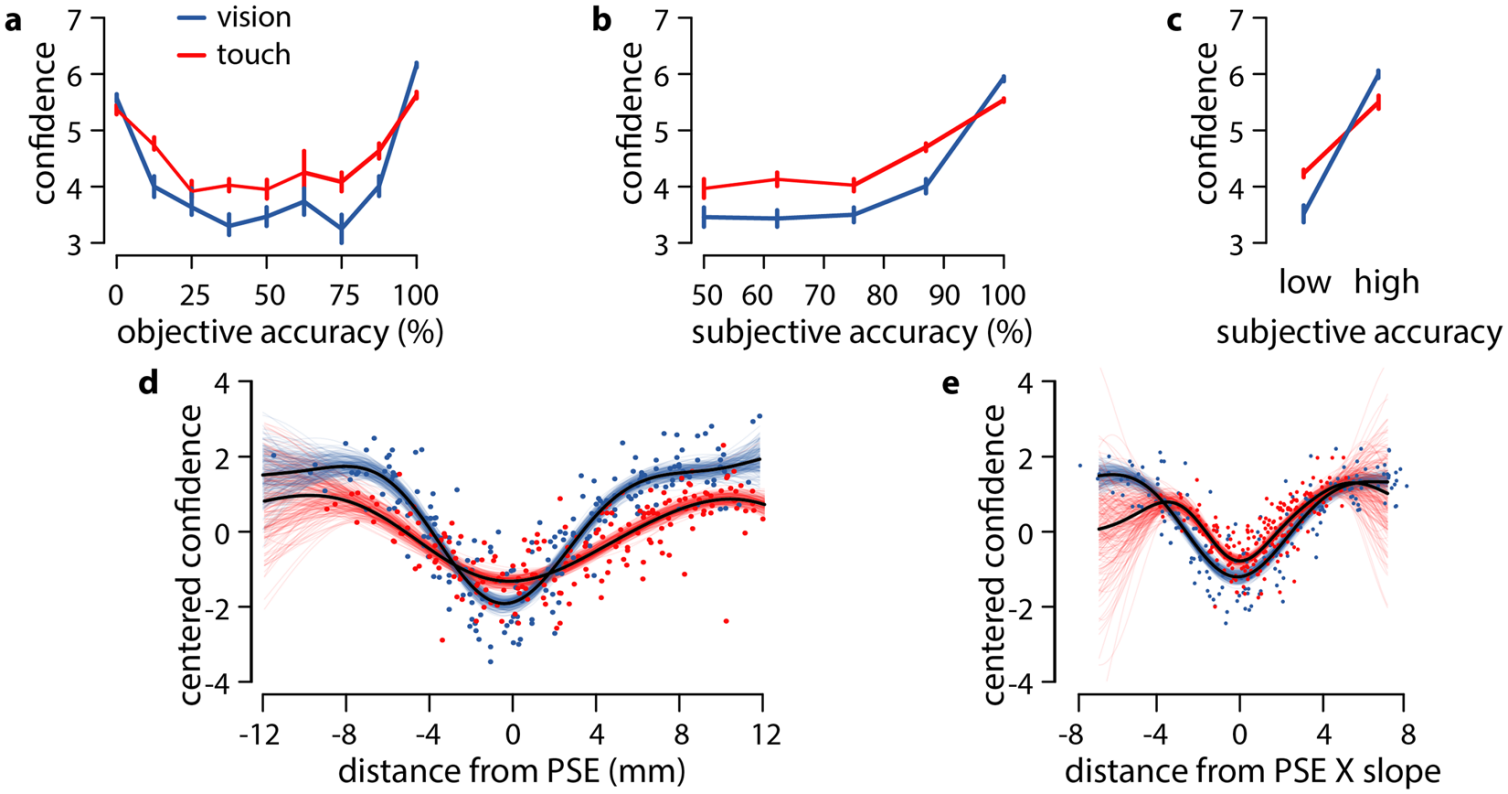
Objective and subjective accuracy. (**a**) Confidence vs. objective accuracy. (**b**) Confidence vs subjective accuracy. (**c**) Subjective accuracy collapsed into low and high levels. (**d**) Confidence ratings on individual trials, relative to that observer’s mean rating, as a function of distance from PSE. Confidence depended on modality, and the distance of the vertical bars’ length from PSE. Confidence is greater in touch for bars close to PSE, and greater in vision for bars far from PSE. Thin lines show samples from the posterior distribution of Gaussian processes describing the relationship between confidence and bar length. Thick lines show maximum a posteriori functions. Confidence is greater in touch than vision for stimuli close to PSE, but greater in vision otherwise. (**e**) Confidence ratings individual trials as a function of normalised distance from PSE - distance from PSE, multiplied by that participant’s psychophysical slope in that modality. Confidence is higher in touch than vision for stimuli within 3 standard deviations of PSE.

In studying metacognition, confidence ratings must be compared across equivalent levels of accuracy. Since observers’ perceptual sensitivity and the strength of the illusion differed between modalities, it was not possible to match observer accuracy directly with the stimuli. We therefore compared confidence for those stimuli which produced equally accurate responses, a posteriori for each participant. A perceptual decision was said to be objectively accurate if the vertical bar was correctly perceived to be longer or shorter than the horizontal bar, given its objective size. A decision was said to be subjectively accurate if the vertical bar was perceived to be longer or shorter relative to the observer’s PSE for that modality. Thus, subjective accuracy was a measure the internal consistency of an observer’s responses. Each stimulus was presented eight times within each modality, and so for each stimulus, within each modality, we calculated observers’ mean subjective accuracy, objective accuracy, and confidence. Figure 3a shows observers’ mean confidence as a function of their objective accuracy. Because of the effect of the Vertical-Horizontal illusion, observers were highly confident for stimuli on which they were consistently correct (when the vertical bar was in fact longer than the horizontal bar, or when the vertical bar was short enough to overcome the illusion) as well as when judging the size of stimuli on which they were consistently incorrect (typically when the vertical was shorter than the horizontal but appeared to be longer). With the stimuli for which the observers were sometimes accurate and sometimes inaccurate, however, confidence was at its lowest. Therefore, the illusion disrupted the usual monotonic relationship between objective accuracy and confidence.

Confidence, however, was monotonically related to subjective accuracy (Fig. 3b). The observers were more confident when presented with stimuli that elicited responses which were more consistent relative to their individual PSEs. Importantly, this relationship interacted with modality, such that confidence was higher in vision for stimuli that elicited subjectively accurate responses, but higher in touch for those stimuli that elicited lower subjective accuracy - that is, for stimuli for which observers responded inconsistently. This effect is shown more clearly in Fig. 3c by collapsing the levels of lower subjective accuracy (less than 99% correct) and comparing them to those of high accuracy (100% correct). We subjected these data to a 2 × 2 (ambiguity × modality) ANOVA, and found a significant main effect of ambiguity on confidence, F(1, 22) = 161.00, p < 0.001, η^2^ = 0.49, and an interaction between ambiguity and modality, F(1, 22) = 72.88; p < 0.001, η^2^ = 0.09, but no main effect of modality, F(1, 22) = 0.394, p > 0.5, η^2^ < 0.001. Post hoc t-tests showed that confidence was significantly higher in touch than vision for the ambiguous stimuli, t(22) = 3.487, p = 0.002, and higher in vision than touch for the non-ambiguous stimuli, t(22) = 2.844, p = 0.009.

This pattern of results more clearly by considering Fig. 3d. This shows observers confidence on individual trials (relative to their mean confidence across all trials, collapsing across modalities) as a function of the distance of the vertical bar from PSE. We quantified this relationship by fitting Bayesian Gaussian processes models, separately for each modality. These models revealed a pattern akin to that sketched in Fig. 2. Close to PSE (from −2.6 mm to +1.9 mm), where the stimuli were perceptually ambiguous, confidence was indeed higher for touch than for vision. Outside of this range, however, where one bar clearly appeared to be longer than the other, confidence was higher for vision than touch. Finally, Fig. 3e shows the same confidence ratings as a function of distance of the vertical bar from PSE normalised by that participant’s sensitivity in that modality. In other words, the x-axis shows the distance of the stimulus from PSE in units of perceptual sensitivity, rather than in millimeters. Stimuli that fall on the same x-axis position here are therefore empirically matched for how difficult they are to discriminate. This shows that the participants’ are more confident in touch than in vision for stimuli from −3.8 SD below PSE to +5.6 SD above it, The finding that average confidence was higher for vision than touch was driven by the presence of stimuli that were extremely far from PSE for vision (>4 SDs), but not for touch, where perceptual sensitivity was lower, and as a result the SDs larger.

In an additional analysis, we estimated the sensitivity of observers’ confidence judgements to the subjective accuracy of their perceptual responses, by fitting meta-d’ signal detection theoretic models to each observer’s data (see Supplementary Material). Mirroring our analysis above, observers’ perceptual judgements were more sensitive in vision, d’ = 3.85, SD = 1.0, than in touch, d’ = 2.41, SD = 0.8, t(22) = 6.490, p < 0.001, d = 1.4. By basing our analyses on observers’ subjective, rather than objective accuracy, their perceptual bias due to the illusion is eliminated. The sensitivity of observers’ confidence ratings to changes in their subjective accuracy was also significantly greater for vision, meta-d’ = 3.49, SD = 1.29, than touch, meta-d’ = 2.14, SD = 0.8, t(22) = 4.524, p < 0.001, d = 0.9. The overall bias in confidence reports did not differ between vision, meta-C = 0.35, SD = 0.20, and touch, meta-C = 0.35, SD = 0.73, t(22) < 0.7, p > 0.5, d = 0.002. Observers’ m-ratios - the ratio of their confidence sensitivity to their perceptual sensitivity, did not differ between modalities, t(22) = −0.645, p > 0.5, d = 0.1, BF_01_ = 3.79, and did not differ significantly from 1 for either modality, t < 1.1, p > 0.3, d < 0.22, BF_01_ > 2.8, indicating that although observers had access to better perceptual information in vision than in touch, the way they used this information in their confident judgements was close to optimal, and did not differ between the modalities. Individual differences in none of these measures were significantly correlated across modalities, r < 0.33.

**Figure 4:**
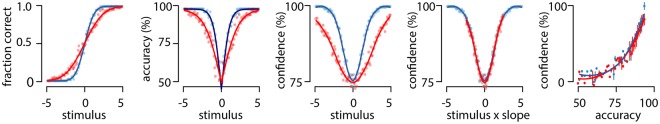
Simulated results from an ideal observer model. Modelled with greater perceptual noise in touch (***σ*** = 2) than in vision (***σ*** = 1), the observer shows a shallower psychometric function for touch (**a**,**b**), and as a result, confidence for touch is usually below that for vision, for a given stimulus (**c**). Unlike observers, however, this ideal observer is equally confident (75%) in both modalities for stimuli at PSE (**c**), and for each stimulus, average confidence tracks average accuracy in the same way across modalities (**d**). Therefore, differences in sensitivity between the modalities alone cannot explain our results.

Finally, is it possible that our results are simply due to differences in perceptual or metacognitive sensitivity between the two modalities? As noted above, participants had greater perceptual sensitivity in vision than in touch, as shown in both the psychometric fits and the SDT analysis. Metacognitive efficiency was close to 1 for both modalities, and as a result type 2 sensitivity was similarly higher for vision than touch. It seems intuitively possible that lower confidence might be reported at PSE in vision, where observers could clearly see that the stimulus was at PSE, than in touch, where observers may not realise they were being presented with particularly difficult stimuli. To formally rule out this possibility, we simulated data from an ideal Bayesian observer, with greater perceptual uncertainty in touch (***σ*** = 2) than vision (***σ*** = 1) (Fig. 4; see Methods, and Supplementary Materials for details). This revealed that while an unbiased observer shows less confidence in vision than in touch for stimuli far from PSE, their confidence for stimuli at PSE averages 75% in both modalities, and confidence tracks accuracy in the same way in each. This is consistent with previous formal work^31,32^ showing that optimal Bayesian observer average reports 75% confidence on trials where there is neutral evidence (that is, for stimuli at PSE). Therefore, our results are not due to differences in perceptual or metacognitive sensitivity between the two modalities but reflect differences in how confidence is computed and reported in each.

## Discussion

Observers could perform the size estimation task set up by the illusory stimuli using either vision or touch but showed higher perceptual sensitivity in vision. As a result, they reported greater confidence overall in their visual judgements, as predicted by the normative models of metacognition. Owing to the bias produced by the illusion, however, confidence ratings tracked objective accuracy poorly, but were monotonically related to subjective accuracy - the extent to which each decision was consistent with the rest of that observer’s judgements. Our results reveal that confidence follows subjective consistency in an illusory context. Consistent with the report of Fitzpatrick *et al*.^7^, confidence was lowest at the category boundary, where the decision between ‘longer’ and ‘shorter’ was most difficult to make. Most importantly, however, confidence ratings did not respect a common mapping for both modalities. Instead, observers were more confident in touch than vision for stimuli to which they responded inconsistently (situations of ambiguity, around PSE), but more confident in vision than touch for stimuli to which they responded consistently. Thus, our results revealed what we might call a “Doubting Thomas effect”: when faced with ambiguous and confusing evidence, the act of exploring an object by touch gives an observer more confidence than vision.

There is now a substantial body of research exploring the nature of confidence in perceptual and cognitive tasks, using confidence ratings^33^, no-loss gambling^34^, post-decision wagering and other behavioural tasks^35,36^. These demonstrate that human and non-human observers can metacognitively access the reliability of their perceptual representations and adjust their confidence accordingly. Along with common currency accounts, which show that confidence acts as a comparison of reliability across different decisions^22,24^, it can be assumed that confidence should be exchanged using the same metric across sensory modalities. A common mapping would provide the most direct way in which an individual can decide which of two decisions reached through different senses is worthier of trust, even when they are separated in time. Moreover, a modality-independent, uniform scale of confidence should enable the optimal aggregation of different sensory estimates for an individual^25^, as it does across agents^37,38^. However, the present results suggest that confidence does not use a consistent metric across different levels of ambiguity across touch and vision.

The present study is one of only a few to explore confidence in touch^39–42^ and it is the first to report that confidence operates differentially in touch and in vision in a matched perceptual task. It invites one to consider what might be special about touch and explain this difference. At the task level, the size estimation task is certainly not accomplished in the same way in touch and vision. To reach a decision by touch, observers swiped across the object when assessing the relative sizes of the two bars, which took a few seconds. With vision, they could acquire evidence about the sizes of the object in a fraction of a second, from a small number of saccades and fixations of very short durations in comparison to comparatively longer hand movements. This difference between the two modalities resulted in distinct patterns of accumulation of evidence over time. Crucially, previous research suggests that, for an equivalent amount of accumulated evidence, temporal differences can affect confidence. Moreover, over protracted perceptual tasks, while the accuracy of decisions is not affected by the time taken to arrive at them, confidence fluctuates^22,43,44^. Nevertheless, in our experiments, temporal factors are unlikely to provide an explanation of the relative overconfidence for touch in ambiguous cases, as these factors would have influenced all decisions uniformly across the whole range of stimuli.

A further difference between the two modalities, and potentially the reason for the observed difference in their confidence profile, may be found in the respective sensorimotor activities. Touch called upon voluntary hand movements to extract size information. In contrast, the visual scan-paths were largely unconscious. Using a visual discrimination task, Fleming and colleagues have identified an action-specific contribution to confidence^36,45^. In the present case, it could also be the case that participants felt more agentive and in control of the evidence collected through haptic exploration rather than vision. Such action-related factors would nevertheless also have affected all decisions uniformly.

Finally, could the results be a consequence of the greater perceptual sensitivity of vision in this task? While this explanation seems intuitively plausible, our simulation results, in line with previous formal proofs^31^, show that this is not the case. Instead, an ideal observer should report the same level of confidence for stimuli as PSE, regardless of their perceptual sensitivity. This counterintuitive result holds because while ideal observer would be more likely to erroneously perceive stimulus that is at PSE to be far above or below it in touch than vision, this would be counteracted by their reduced overall confidence in the less sensitive modality. Therefore, our results are not simply the result of the difference in sensitivity between the modalities. A future challenge will be to reconcile these results with work framing multimodal sensory perception as Bayesian integration, for instance studies investigating sensory fusion and observed visual dominance over touch^46,47^.

The present work speaks to instances in daily life where senses operate serially, and confidence must be compared across sensory domains. Rather than a consistent, single metric, our results suggest confidence might adopt variable metrics across cases. As a possible extension, it would be valuable to investigate whether the effect occurs where observers are asked to make independent decisions, one in vision and one in touch, and explicitly compare the two. If the effect holds, observers would select tactile-based decisions to be those for which they feel comparatively more confident when accuracy is at its lowest in each modality. This suggests new ways in which to test the common currency model, focusing on cases where the same attribute is compared^23,24^. Relative overconfidence in touch would also need to be tested in non-illusory settings. Other tasks might also open the possibility to control for decision time, for differences in performance, and for accuracy through task difficulty. The illusory case employed here however comes closer to explaining why people would use touch when they are in doubt about the properties of an object. Even if it does not provide a better estimate, it provides a higher sense of certainty, something that Esquirol warned of in the field of psychopathology when he observed that “touch, often appealed to by reason to correct the other senses, may also deceive”^48^. Although touch is not always believed more than sight, it is the last to be doubted.

## Methods

### Observers

The experiments were conducted at the Centre for the Study of the Senses, University of London. With both verbal and written instructions, 24 healthy volunteers (11 females and 13 males, age range 21–29) were briefed as to the broad nature of the study. Sample size for this study was determined based on previous research on the VH illusion. Ethics approval for the experiment was obtained from the School of Advanced Study, Research Ethics Committee. Written informed consent was obtained from all participants and the experiment was performed in accordance with the relevant guidelines and regulations of the School of Advanced Study, University of London.

### Experimental design

Testing consisted of a training phase and an experimental phase. Observers were instructed to explore the objects by touch or by vision. Each object consisted of two bars, arranged in an inverted T shape. They were in-house constructed and consisted of a square backing card (polystyrene foam layer sandwiched between clay-coated paper; measuring 15 by 15 cm) with a raised inverted T-shape at the centre. The bars of the T were made of polyvinyl chloride plastic 3 mm half-round stock machined to interlock without a gap. They were sanded down to a very fine roughness (P1200 ISO grit) to enhance their frictional properties and prevent stickiness. For all nine stimuli types, the horizontal bar was 30 mm in size while the vertical bar varied in sizes by 2 mm step between 18 mm and 34 mm (Fig. 1). Suzuki and Arashida^29^ used a horizontal bar of 50 mm as a standard, and a vertical bar varying from 30 to 70 mm by steps of 1 mm as a comparison (41 stimuli). We opted for a shorter standard (30 mm) to minimise arm movements in haptic exploration, and a sparser stimulus set to provide for multiple repeats. The set of values was such that three stimuli would be shorter (18, 20, 22 mm) and three to be longer (30, 32, 34 mm) since the strength of the illusion is of the order of 10%. The three other stimuli (24, 26, 28 mm) would then be ambiguous (less than a 10% difference in size). In the haptic condition, objects were presented at a fixed distance, at arm’s length in front of the observer, lying flat on a table, in such a way that the horizontal bar would correspond to a tangential movement, and the vertical bar to a radial movement (Fig. 2a). The choice of task, that is size estimation under the influence of common perceptual illusion, allowed us to vary ambiguity (the probability of being correct - how sure I am that my judgment is on this side of the criterion), to see the manner in which subjective (and not objective) accuracy tracks confidence across modalities. In the task, observers closed their eyes and did not have to move their arms during exploration. They explored the objects with their index finger in two swipes (only) with a fixed order of radial followed by tangential movements. When exploring the objects visually, the object was presented at arm’s length in front of the observer in the same position as in the haptic condition. In each trial, observers had a five second time window to declare whether the vertical bar was shorter or longer than the horizontal bar. Having given their verbal response, they were prompted to report how confident they were of their choice. Confidence reports were given using a scale from 1 to 7 (from low to high certainty). A training phase of two blocks was administered to familiarise observers with the procedure, one for each modality. Each block comprised 18 pseudo-randomised trials, presenting each of the nine stimuli twice. In the testing phase, visual and tactile blocks comprised 72 pseudo-randomised trials, that is eight repetitions of the presentation of each of the nine stimuli. Modality block order was counterbalanced across observers. The individual portrayed in Fig. 2a was not a study participant and has provided informed consent for publication of identifying images in an online open-access publication.

### Statistical Analysis

Data about size judgements and confidence ratings were analysed using the R software package^49^. Cumulative Gaussian psychometric functions with mean, μ, and standard deviation, σ, were fit for each observer in each modality to quantify the relationship between the length of the vertical bar and the probability of observers responding that it was longer than the horizontal. The μ parameter reflected the PSE—the point at which an observer was equally likely to give either response. The slope, S, reflecting participants’ sensitivity to changes in the stimuli, was computed using 1/σ; thus, the smaller the standard deviation, the more sensitive observers were to changes in the stimuli.

To analyse confidence ratings, we labelled trials as objectively accurate if the observer correctly indicated that the vertical bar was longer or shorter than the 30-mm reference, and as subjectively accurate if they responded ‘longer’ when the vertical was longer than their PSE for the modality. We calculated the observers’ average objective accuracy, subjective accuracy, and confidence for each eight repetitions of each stimulus. Owing to the relatively small number of trials at a sufficiently large number of levels of average subjective accuracy, we collapsed across all trials where subjective accuracy was less than 100% and conducted a two-by-two ANOVA (subjective accuracy: 100% or smaller than 99% vs modality: vision or touch). To capture ambiguity at a more direct perceptual level, we explored the relationship between confidence and the difference between the target size from an observer’s PSE in each modality. This measure quantified the perceptual ambiguity of the stimuli, with stimuli around the PSE being the most ambiguous. To increase the precision of individual observers’ PSE and perceptual sensitivity, we refit the probit model above as a Bayesian hierarchical regression model, using the brms package for R. We used non-informative prior distributions, with fixed effect β ~ Normal(0, 10) and random effect variance σ ~ Half Cauchy(0, 4). We also centred confidence around the mean confidence rating of each observer collapsing across both modalities to compensate for observer-specific systematic overconfidence or under-confidence. In the first model, we tested the relationship between confidence and distance from PSE measured in millimeters. In the second model, we used the distance from PSE multiplied by the probit regression slope for that participant, in that modality. This yields a measure of the distance of the stimulus from PSE in standard deviations, similar to the d’ measure used in signal detection theory. For inference, we fit separate Bayesian Gaussian process models to each modality, using the PyMC3 package for Python^50^. These are non-parametric models that can capture the non-linear relationship between variables bypassing the need to specify a class of link functions. Figure 3d,e show 500 functions drawn from the posterior distribution for each modality, along with the median predicted confidence levels. Supplementary Figs S7–S8 show the posterior difference in confidence between the two modalities, as a function of distance from PSE.

We simulated the performance of a Bayesian optimal observer on an idealised version of our task. The observer was presented with 101 distinct stimuli ranging from −5 to 5, and 100 repetitions of each stimulus in each modality. The values, ${y}_{i}\,$observed in trials, *i*, were contaminated by perceptual noise drawn from a normal distribution, $N({\mu }_{i},\,{\sigma }^{2})$, with mean, ${\mu }_{i}$, set to the true value of the stimulus, and standard deviation, $\sigma$. The standard deviation of the noise was set to 1 for vision and 2 for touch. The observer responded *r* = 1 when *y* > 0, and *r* = 0, otherwise. To rate confidence, the observer used the knowledge of its sensory uncertainty and computed the probability of giving an erroneous response, that is, the posterior probability of *r* = 0 when *r* = 1, and that of *r* = 0 when *r* = 0. The reported confidence, ${C}_{i}$, then was, $1-P(\text{Error}\,|\,\text{abs}({y}_{i}),\,\sigma )$, where, if ${\rm{\Phi }}$ is the Normal cumulative density function,

$$P({\rm{Error}})={\int }_{-\infty }^{0}N(x\,|\,{\rm{abs}}({y}_{i}),\sigma )dx={\rm{\Phi }}(0\,|\,{\rm{abs}}({y}_{i}),\sigma )$$

Further details of this modelling approach, and results from models with additional modality-specific biases, can be found in Supplementary Materials.

## Electronic supplementary material

Supplementary Materials

## Data Availability

The datasets generated during and/or analysed during the current study are available from the corresponding author on reasonable request.
