# Supplementary material for: Confidence is higher in touch than in vision in cases of perceptual ambiguity
Source: Sci Rep. 2018 Oct 23;8:15604. doi: 10.1038/s41598-018-34052-z (PMC6199278; doi:10.1038/s41598-018-34052-z)
Supplement: Supplementary file 1 — Supplementary Materials [file 41598_2018_34052_MOESM1_ESM.pdf]

Confidence is higher in touch than in vision in cases of perceptual ambiguity

Merle T. Fairhurst[1,2], Eoin Travers[1,3], Vincent Hayward[1,4], and Ophelia Deroy[1,2]

[1]Centre for the Study of the Senses, School of Advanced Study, University of London, UK
[2]Munich Center for Neuroscience, Ludwig Maximilian University, Munich, Germany
[3]Institute of Cognitive Neuroscience, University College London, London, UK
[4]Sorbonne Universités, UPMC Univ Paris 06, ISIR, 75005, Paris, France

Supplementary Information

Similarity of the Vertical-Horizontal Illusion in Vision and Touch

The Vertical-Horizontal illusion was previously found to function in a similar fashion across vision and touch (Suzuki and Arashida, 1992) despite some differences in the perceptual sensitivity and in the magnitude of the illusion. Our results show that, on average, with vision the vertical bar appeared to be 1.2 times longer than the horizontal bar. With touch, this ratio was 1.3. Thus, the magnitude of the illusion was slightly greater for touch than for vision. These values, however, were in line with what has been reported previously by Suzuki and Arashida (1992).

Supplementary Results

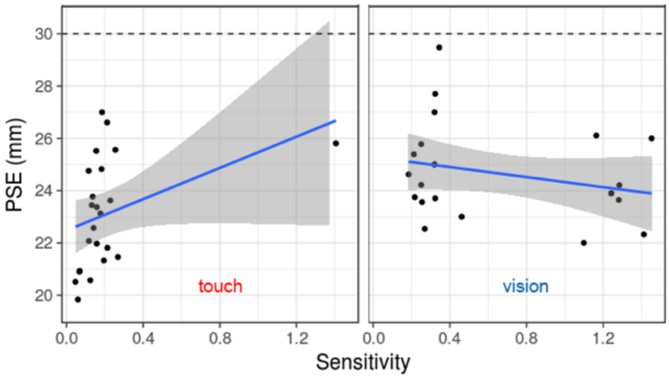

Figure S1 – Observers who were more sensitive to changes in the stimuli were not significantly less susceptible to the Vertical- Horizontal illusion (showed PSEs closer to 30 mm) in touch, r(21) = -.38, p = .073, BF = 1.02, or in vision, r(21) = .27, p = .217, BF = 0.46, although excluding one participant with high sensitivity for touch revealed a significant negative relationship, r(21) = -.50, p = .018, BF = 3.04. Error bars show regression standard error.

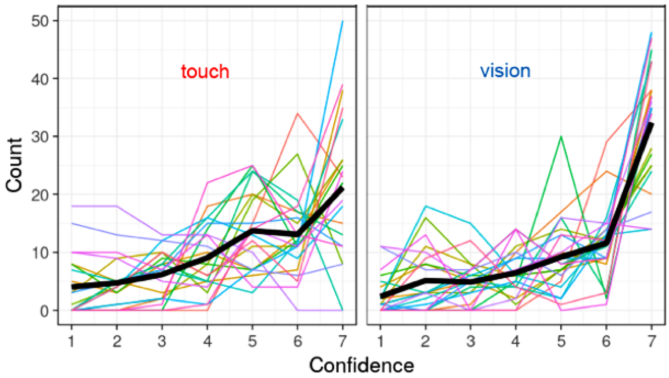

Figure S2 – Confidence ratings in each modality. Coloured lines show distributions for each observer. Black lines show averaged ratings.

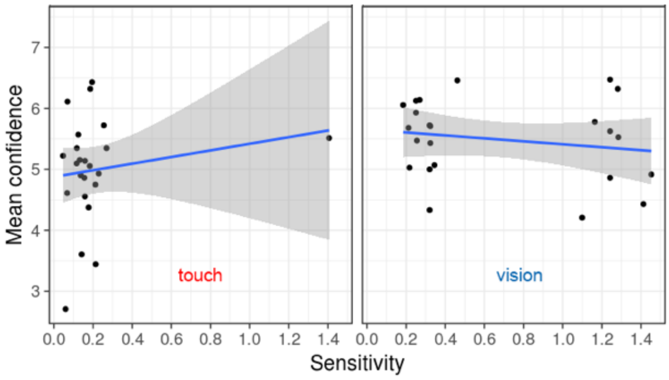

Figure S3 – There was no significant relationship between observers' perceptual sensitivity in each modality and their average confidence for trials in that modality, in either touch: $r(21) = .17$, $p = .450$, $\text{BF} = 0.30$, or vision, $r(21) = -.18$, $p = .406$, $\text{BF} = 0.31$. This conclusion was not affected by removing the outlier for touch.

Signal Detection Analysis

Signal detection theoretic (SDT) analyses have become commonplace in studies of metacognition for perceptual decisions (Fleming and Lau, 2014). The meta-d' statistic (Maniscalo and Lau, 2014) provides an index of observers' sensitivity to changes in the accuracy of their responses (their level 2 sensitivity), in the same way, and on the same scale, as conventional d' indexes their sensitivity to changes in the stimulus (level 1 sensitivity). Because of the strength of the VH illusion, however, there were few trials where the vertical line was longer than the horizontal, and almost none where it was longer but observers stated that it was shorter (misses), and so it was not possible to fit a standard signal detection model. Instead, we used each observer's modality-specific PSE to define trials as hits (the vertical bar was longer than PSE and the observer responded 'longer'), *misses* (responses of 'shorter' for the same stimuli), *correct rejections* (vertical was shorter than PSE, and observer responded 'shorter'), and *false alarms* (vertical was shorter, observer responded 'longer'). Thus, our analysis eliminates differences in level 1 response bias between observers, but provides an estimate of their sensitivity to variations in the stimuli, and the consistency of their responses. All seven possible confidence ratings were included as possible level 2 responses in the analysis. We estimated response (level 1) and confidence (level 2) SDT parameters separately for each observer, within each modality, using MATLAB scripts made available by Maniscalo and Lau (2014; `http://www.columbia.edu/~bsm2105/type2sdt/`).

Figure S4 shows estimates for d' (level 1 sensitivity), meta-d' (level 2 sensitivity), and m-ratio (the ratio of d' to meta-d', with a value of 1 indicating that the same information was used for perceptual judgements and for confidence ratings. A 2 (modality: touch or vision) X 2 (level: d' or meta-d') repeated measures ANOVA indicated that sensitivity was higher for touch than for vision, $F(1, 22) = 136.92$, $p < .001$, $\eta^2 = 0.34$. There was no difference between d' and meta-d', $F(1, 22) = 1.79, p = .195$, $\eta^2 = 0.03$, and no modality $\times$ level interaction, $F(1, 22) < 0.1, p > .8, \eta^2 < 0.01$. The m-ratio did not differ significantly between modalities, t(22) = 0.645, p ¿ .5, BF = 0.27, and was not significantly different from 1 for vision, $t(22) = 0.072, p > .9, \mathrm{BF} = 0.22$, or for touch, $t(22) = 1.021$, $p = .318$, $\mathrm{BF} = 0.35$. Therefore, this analysis indicates that vision provides more information than touch in this task, and observers generally lost little of this information between giving their responses and their confidence ratings. Importantly, while metacognitive sensitivity was poorer in touch than in vision, this difference was not greater than would be expected given a reduced perceptual sensitivity for touch.

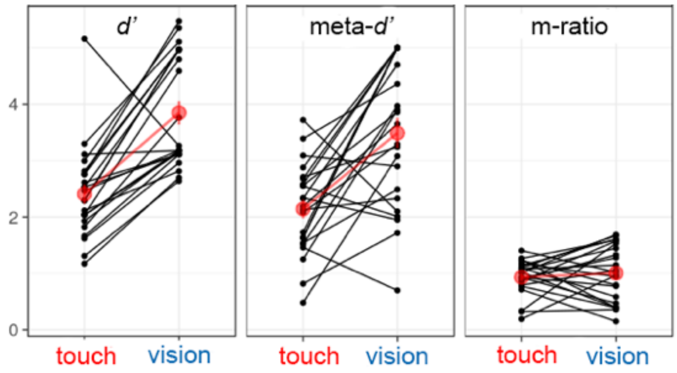

Figure S4 – Signal detection parameters, by modality. Individual observers' parameters are shown in black. Sample mean and standard errors are shown in red.

Gaussian Process Analysis

We also explored how observers' confidence ratings changed as a function of the difference between the stimuli and observer's PSEs, in that modality. Figure S5 plots mean confidence ratings against the distance of the vertical bar from PSE (in 2 mm bins), aggregated across stimuli, and across observers. As would be expected given the greater perceptual sensitivity for vision in this task, confidence was higher in vision than in touch for most stimuli. However, consistent with the 'doubting Thomas effect', confidence was higher for touch for stimuli closest to PSE.

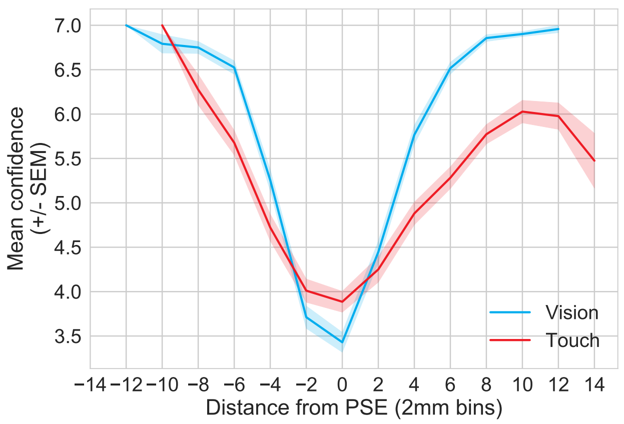

Figure S5 – Confidence was generally higher for vision than for touch, except for stimuli close to PSE, where confidence was lowest overall, but higher for touch than vision (the 'doubting Thomas effect').

A natural approach to analysing these data would be to conduct a 2 (modality) × 2 (ambiguity: close to or far from PSE) ANOVA. However, as we had not defined in advance how close stimuli would be to PSE before confidence in touch should be greater than that in vision, we instead fit a Bayesian model to estimate relative levels of confidence in each modality as a continuous function of distance from PSE. To do so, we first centred each observers' confidence ratings around their overall mean, to eliminate individual differences in baseline confidence ratings. We then fit separate Bayesian Gaussian process models to the data from each modality, using uninformative squared exponential covariance matrices. These models were fit using the `pymc3` package for python, and the code used can be found along with the data set at `https://osf.io/yseun/`. Figure S6 (also Figure 3d) shows 500 functions sampled from the posterior distribution for each modality, along with the median predicted confidence levels. These posterior samples allow us to make probabilistic inferences about the extent of the 'doubting Thomas effect'. For instance, according to the most likely estimate, confidence is expected to be higher in touch than in vision for stimuli between -1.9 mm and +2.7 mm from PSE, and we can be 95% certain that confidence should be higher in touch for stimuli between -1.3 mm and +2.0 mm from PSE.

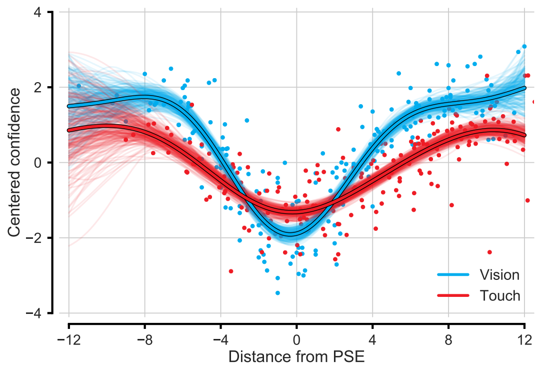

(a) Confidence as a function of distance from PSE.

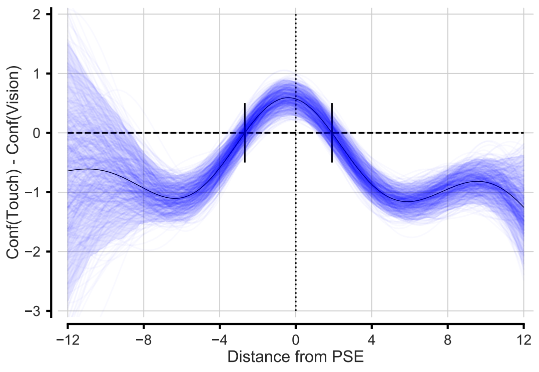

(b) Difference in confidence as a function of distance from PSE.

Figure S6 – **(a)** Bayesian Gaussian processes fit to model centered confidence as a function of the distance of the stimulus from PSE, separately for each modality. **(b)** Posterior difference between the two modalities, as a function of distance from PSE. Confidence is predicted with 50% certainty to be lower in vision than in touch for stimuli between -1.9 mm and +2.7 mm from PSE (vertical lines), and with 95% certainty for stimuli between -1.3 mm and +2.0 mm from PSE.

A limitation of this analysis, however, is that perceptual sensitivity is not matched between the two modalities. As a consequence, a touch judgement about a stimulus 2mm longer than the PSE for touch is more difficult than a visual judgement about a stimulus 2mm longer than the PSE for vision. To address this, we normalised the distance of the stimuli from PSE using the probit regression for each participant in that modality. This yields a metric of the distance of the stimulus from PSE in perceptual standard deviations. This means that trials using stimuli matched for normalised distance from PSE are also matched for difficulty. If confidence does act as a common currency between modalities, confidence ratings in each modality should be equal for a given distance from

PSE. Figure S7 (also Figure 3e) shows the analysis above, repeated using this standardised metric. This confirms that even when controlling for objective perceptual difficulty, confidence is higher in touch than in vision for stimuli close to PSE. Because sensitivity is lower in touch, there are few touch stimuli more than 4 SD from PSE. Confidence ratings for vision stimuli in this range were very high, reflecting the ease of these judgements.

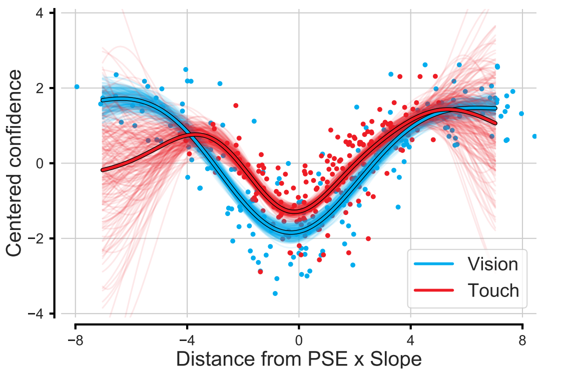

(a) Confidence as a function of normalised distance from PSE.

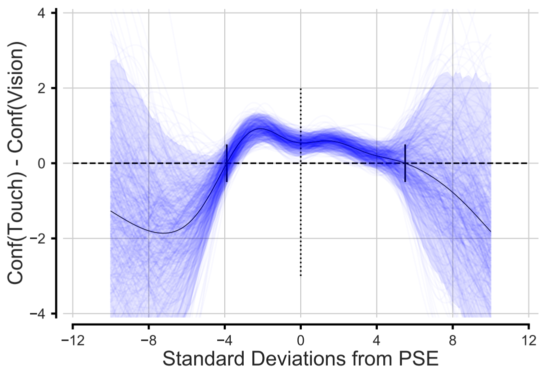

(b) Difference in confidence as a function of normalised distance from PSE.

Figure S7 – **(a)** Bayesian Gaussian processes fit to model centered confidence as a function of the normalised distance of the stimulus from PSE — distance in millimetres multiplied that participant's psychometric slope in that modality – separately for each modality. **(b)** Posterior difference between the two modalities, as a function of normalised distance from PSE. Confidence is predicted with 50% certainty to be lower in vision than in touch for stimuli between -3.8 SD below PSE to +5.6 SD.

Bayesian Model of Confidence Bias Toward Touch for Ambiguous Stimuli

In this section we test, using an ideal observer model, whether the reported metacognitive effect of preferential confidence in touch over vision in the case of ambiguous stimuli could be due to differences in perceptual sensitivity between the two modalities. The hypothesis could be that a high accuracy in vision lets observers better detect the ambiguous character of the stimuli that are near to a point of subjective equivalence. High perceptual accuracy could then lead observers to lend low confidence to judgements about those stimuli that are clearly ambiguous. Conversely, lower accuracy in touch would lead observers to fail to realise that some stimuli are close to a point of subjective equivalence, thus lending too much confidence to judgements about these undetected ambiguous stimuli. This hypothesis is consistent with our psychophysical data. Observers had greater perceptual sensitivity in vision than in touch, see main Figs. 1d-e, and in both modalities observers had overall more confidence in vision than in touch, see main Fig. 3a.

We simulated the performance of a Bayesian optimal observer on an idealised version of our task. The observer was presented with 101 distinct stimuli ranging from -5 to 5, and 100 repetitions of each stimulus in each modality. The values, y_i observed in trials, i, were contaminated by perceptual noise drawn from a normal distribution, $\mathcal{N}(\mu_i, \sigma^2)$, with mean, μ_i, set to the true value of the stimulus, and standard deviation, σ. The standard deviation of the noise was set to 1 for vision and 2 for touch. The observer responded $r = 1$, when $y_i > 0$, and $r = 0$, otherwise. To rate confidence, the observer used the knowledge of its sensory uncertainty and computed the probability of giving an erroneous response, that is, the posterior probability of $y_i < 0$ when $r = 1$, and that of $y_i > 0$ when $r = 0$. The modelled confidence, C_i, then was, $1 - P(\text{error}|\mu_i, \sigma)$ where if Φ is the normal cumulative density function,

$$P(\text{error}|y_i, \sigma) = \int_{-\infty}^{0} \mathcal{N}(x|\text{abs}(y_i), \sigma)dx = \Phi(0 \mid \text{abs}(y_i), \sigma) \tag{1}$$

Figures S8a and S8b show the results generated by the ideal observer.The psychometric function has, indeed, a shallower slope for touch than for vision. The model successfully predicts lower overall confidence ratings for touch, Fig. S8c. Crucially, however, the model fails to predict greater confidence in touch for ambiguous stimuli, Fig. S8c, and confidence as a function of normalised distance from PSE (distance $\times$ slope) is identical in both modalities, Fig. S8d-e. The predicted confidence for ambiguous stimuli is the same in the two modalities. It is in line with the result of Hangya, Sanders and Kepecs (2016), i.e. 75%, and confirms the predictions of a common currency hypothesis because confidence tracks accuracy.

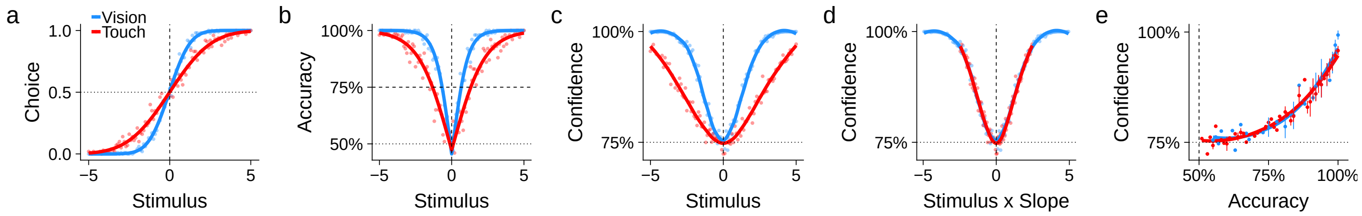

Figure S8 – **a**) Responses as a function of the stimulus. **b**) Response accuracy. **c**) Confidence ratings as a function of the distance of the stimulus from PSE. Note minimum at 75%. **d**) Confidence ratings as a function of the normalised distance of the stimulus from PSE. **e**) Average confidence plotted against average accuracy over 100 repetitions for each stimulus.

The empirically obtained pattern of results can be partly reproduced by biasing confidence ratings. Figure S9 shows the predictions of an observer which, as before, is more sensitive with vision than with touch but which estimates confidence assuming that $\sigma = 1$ for both modalities. This model predicts higher confidence in touch for ambiguous stimuli, Fig. S9c, even when confidence is plotted as a function of normalised distance from PSE, Fig. S9d. However, it fails to fully explain the empirical results since it predicts that for a given level of difficulty, confidence in touch should be always greater or equal to that in vision, Fig. 9d-e.

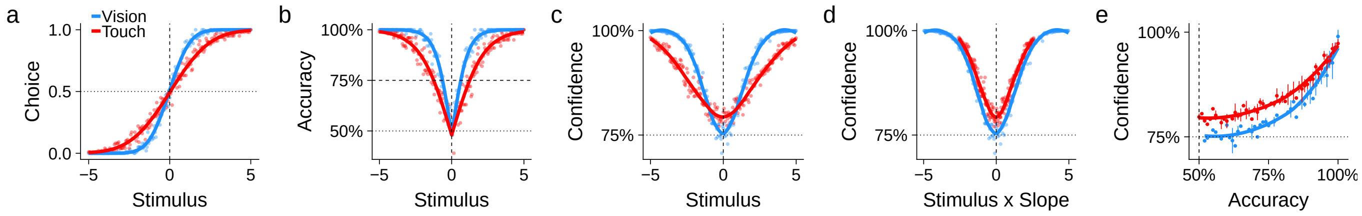

Figure S9 – Responses **a**) and accuracy **a**) as a function of the stimulus, as above. **c**) Confidence is boosted in touch, particularly close to PSE. **d**) This boost holds when controlling for normalised distance from PSE. **e**) As a result, confidence at a given level of accuracy is higher in touch than vision.

References

Fleming, S. M. and Lau, H. C. (2014). How to measure metacognition. *Frontiers in Human Neuroscience*, 8:443.

Maniscalco, B. and Lau, H. (2014). Signal detection theory analysis of type 1 and type 2 data: meta-d?, response-specific meta-d?, and the unequal variance SDT model. In *The cognitive neuroscience of metacognition* (pp. 25–66). Springer.

Suzuki, K. and Arashida, R. (1992). Geometrical haptic illusions re- visited: Haptic illusions compared with visual illusions. *Perception & Psychophysics*, 52:329–335.

Hangya, B., Sanders, J. I., and Kepecs, A. (2016). A mathematical framework for statistical decision confidence. *Neural Computation*, 28(9):1840–1858.
